# Nitrogen addition and drought impose divergent effects on belowground bud banks of grassland community: a meta-analysis

**DOI:** 10.3389/fpls.2024.1464973

**Published:** 2025-01-06

**Authors:** Jing Wu, Xian-zhang Hou, Jin-lei Zhu, Ren-hui Miao, Michael Opoku Adomako

**Affiliations:** ^1^ School of Life Sciences, Taizhou University, Taizhou, China; ^2^ Institute of Wetland Ecology & Clone Ecology/Zhejiang Provincial Key Laboratory of Plant Evolutionary Ecology and Conservation, Taizhou University, Taizhou, China; ^3^ Chinese Academy of Forestry Research, Institute of Forestry, Beijing, China; ^4^ Dabieshan National Observation and Research Field Station of Forest Ecosystem at Henan, International Joint Research Laboratory for Global Change Ecology, School of Life Sciences, Henan University, Kaifeng, China

**Keywords:** aboveground productivity, anthropogenic disturbances, belowground bud banks, clonal growth, clonal organs, global change

## Abstract

**Introduction:**

Belowground bud banks (or bud-bearing organs) underlie grassland regeneration and community succession following ecosystem perturbations. Disturbances of nitrogen (N) enrichment, overgrazing, wildfire, and drought substantially affect grassland ecosystem succession and aboveground productivity.

**Methods:**

To understand the magnitude and direction of the disturbances on the belowground bud banks, we conducted a meta-analysis on 46 peer-reviewed studies published from 1980 to 2023. The meta-analysis comprises 231 observations of bud bank density per unit area and 410 observations of bud bank density per tiller.

**Results:**

Results indicate that N addition remarkably promotes bud banks densities and plant functional groups of grass in the belowground bud banks. While drought negatively affects bud banks densities and functional groups of grasses and forbs. We found that effects of the N addition and drought on the bud banks depend on the bud type, e.g., root sprouting buds, bulb buds, and dormant buds. However, grazing and wildfire have no significant effect on the bud banks.

**Discussion:**

Our results suggest that the N addition and drought may significantly exert promotional and inhibitory effects, respectively, on belowground bud banks, critically altering plant regrowth, community succession, and grassland community dynamics.

## Introduction

Globally, grassland communities are increasingly faced with disturbances, including nitrogen (N) addition, overgrazing, wildfire, and drought, critically underlying the loss of grassland community stability (Dawson et al., 2011; Pecl et al., 2017; Schulte to Bühne et al., 2021). Grassland ecosystems provide crucial ecosystem functions and services despite being very sensitive to the disturbances (Luo et al., 2023). This underscores the significance of belowground bud banks, serving as ecological insurance for grassland recovery and community succession following periods of environmental perturbation (Hoover et al., 2014; Ott et al., 2019; Qian et al., 2023; Wu et al., 2024). Although these environmental stressors critically impose damaging effects on individual plant species, their sensitivity and response to these effects may differ among plant functional types, including grasses and forbs (Qian et al., 2022; Song et al., 2011; Wang et al., 2019). However, it remains unclear how such disturbances – N addition, drought, grazing, and wildfire − affect belowground bud banks and the mechanisms driving such impacts.

Belowground bud banks are commonly associated with a suite of bud-bearing organs (e.g., rhizomes, tillers, and ramets) and the capacity to balance resource allocation to ensure the growth, stability, and maintenance of plant populations and communities (Klimešová et al., 2023; Qian et al., 2021; Wu and Yu, 2022). Belowground bud banks also represent a pool of carbohydrate storage structures tightly linked with their resilience and capacity to resprout under favorable environmental conditions (Klimešová et al., 2021; Ru et al., 2023). Given the frequent droughts, wildfires, and grazing in grassland ecosystems, belowground bud-bearing organs remain crucial for such ecosystems for their ultimate aboveground regrowth following the period of perturbation (Donovan et al., 2020; Twidwell et al., 2016). For example, Donovan et al. (2020) found no evidence of a persistent wildfire in North America’s grassland biome due to the rapid regrowth of all vegetation functional types, suggesting the importance of active belowground bud banks. On the contrary, Twidwell et al. (2016) observed that extreme drought following a period of wildfire significantly decreased the resprouting densities of woody shrubs and aboveground recruitment by 35−55% compared to areas that did not burn in the southern Great Plains of North America. This suggests that belowground bud-bearing organs represent a vital determining factor for aboveground recruitment and regeneration rate (Ott et al., 2019; Qian et al., 2021). However, such attributes of belowground bud banks can be constrained by serious disturbances, with cascading negative impacts on ecological restoration and community succession (Klimešová et al., 2021).

Indeed, serious disturbances negatively affect grasslands via a decrease in the density and regeneration capacity of belowground bud banks (Fischer and Knutti, 2014; Ru et al., 2023). Such impacts on grassland ecosystems have been reported at regional and global scales (Ciais et al., 2005; Leys et al., 2018; Zhao and Running, 2010). In Europe, for instance, an intense drought-induced decline in net primary productivity in 2003 has been reported (Ciais et al., 2005), while a global-level decrease in terrestrial primary productivity caused by drought between 2000 and 2009 has been documented (Zhao and Running, 2010). Prolonged impacts of these disturbances have caused the degradation of many temperate grasslands in Asia and North America and tropical grasslands in South America and Africa (Bardgett et al., 2021; Stevens et al., 2004). It is worth noting that these disturbances complement each other, thereby maximizing their gross impacts on ecosystems. For instance, chronic N additions have been found to exacerbate drought effects on grassland productivity (Meng et al., 2021). Across many field and controlled studies, variation in individual plant vulnerability has been implicated as the key limiting factor for ecosystem recovery after these disturbances (Debinski et al., 2010; Qian et al., 2023; Wang et al., 2012; Xu et al., 2021). One reason for such variation could relate to the differential responses among plant functional types, especially grasses and forbs (Qian et al., 2023; Xu et al., 2017). As a confirmation, Qian et al. (2023) have reported a consistent decrease in the density of the belowground bud bank of forbs but not grasses in response to drought. Besides the individual differences, however, mechanisms underlying such variation in plant functional type responses to disturbances, including N addition, drought, grazing, and wildfire, remain inadequate.

Understanding such disparities in bud bank responses among plant functional types is crucial for predicting future climate and human-derived impacts on grassland communities. Belowground bud banks of different plant functional types may vary in their responses to environmental stress (Carter et al., 2012; Dalgleish and Hartnett, 2009; Klimešová and Klimeš, 2007; Zhao et al., 2019). Therefore, plant functional types well-adapted to a given disturbance may exhibit a more pronounced regrowth after periods of disturbance (Hoover et al., 2014; Mackie et al., 2019). Most previous studies have demonstrated that grasses often show higher resistance to intense drought and grazing owing to their resource-use strategies compared to forbs (Carter et al., 2012; Xu et al., 2021, 2017). In an experimental study, forbs exhibit less resistance to long-term drought than grasses. However, the belowground organs of forbs had the quickest recovery rate in that study (Carter et al., 2012). While an annual wildfire least affected the belowground bud bank of grasses, it remarkably decreased that of forbs by 125% (Dalgleish and Hartnett, 2009). These differential responses of plant functional types are relevant for understanding ecosystem-level consequences of plant communities, especially those ecosystems that are dominated by a peculiar functional type.

The duration of occurrence and intensity of a disturbance regime primarily modulate the severity of impacts driven by climate change and human activities (Tonkin et al., 2017; White and Hastings, 2020). While an extreme drought condition is tightly linked with frequent and intense wildfires (Chikamoto et al., 2017; Pontes-Lopes et al., 2021; Wragg et al., 2018), increasing N addition promotes the growth of grasses and modifies their palatability, ultimately determining grazing preference and intensity. Therefore, we hypothesize that environmental stressors, including N addition, drought, grazing, and wildfire, may impose divergent effects on belowground bud banks and that such differences may vary depending on the severity, bud types, and plant functional types.

We conducted a meta-analysis of existing studies to test these hypotheses and specifically asked whether (1) disturbances of N addition, drought, grazing, and wildfire affect belowground bud bank densities in similar ways; (2) differences in plant functional types mediate belowground bud banks’ responses to the disturbances; (3) bud type differences mediate belowground bud banks’ responses to the disturbances. It was predicted that N addition and drought effects on bud banks may exhibit divergent patterns in many prominent ecosystems, e.g., grasslands.

## Materials and methods

### Data compilation

**Figure 1:**
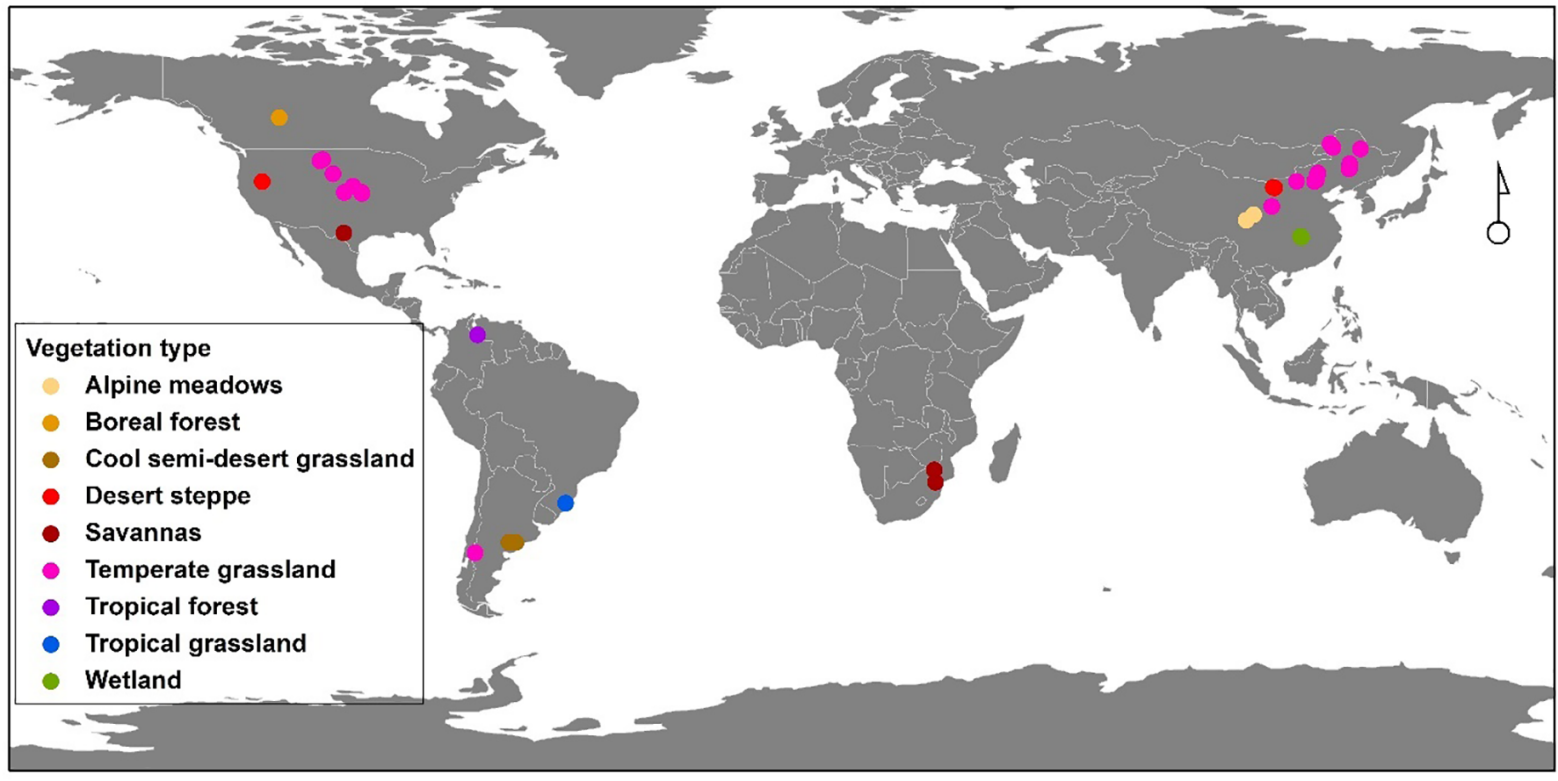
The biomes and study sites over the world covered in the analyzed dataset.

We compiled data from studies that have reported belowground bud bank responses to N addition, drought, grazing, and wildfire disturbances by conducting a literature search for peer-reviewed publications in the Web of Science (http://apps.webofknowledge.com/) and Google Scholar. We used the following search string: ‘climate change’ OR ‘global change’ OR ‘human disturbance’ OR ‘drought*’ OR ‘N addition’ OR ‘increased precipitation’ OR ‘fire’ OR ‘grazing’ OR ‘clipping’ OR ‘herbivory’ AND ‘buds’ OR ‘bud bank’ OR ‘bud density.’ All published records from 1980 to 2023 were included in the search. We then screened all the studies for publications that met the criteria: (i) the publication reported effects of manipulating at least one of the following disturbances − N addition, drought, wildfire, and grazing − as well as clipping on bud bank densities of the whole plant community and/or different plant functional types; (ii) the publications that reported mean values, sample sizes, and variances for bud bank densities. In total, 46 publications met the criteria (see Materials and Methods in S1), with 246 observations on the bud bank density per unit area, 410 observations on the bud bank density per tiller, and 174 observations for the wildfire moderator, 281 observations for the grazing moderator, 149 observations for the drought moderator, as well as 52 observations for the N addition moderator. The biomes and study sites of all 46 publications across the world covered in this dataset are shown (**Figure 1**). Also, detailed information on studies, including classification of disturbances, ecosystem, plant functional type, and bud type, as well as the disturbances and the subsequent effects are shown in the **Supplementary Material** (**Supplementary Table S1**).

We extracted mean values of the bud bank density and their corresponding variances (standard deviations, standard errors, or 95%-confidence intervals) and sample sizes directly from the text, tables, or figures using IMAGE J 1.47 v (Rasband, 2013). For the studies involving N addition, water addition, wildfire, grazing, and/or clipping, we considered the ambient level (i.e., no treatment) as the ‘control’ and ‘treatment’ for level(s) such as the N addition, water addition, wildfire, grazing, and/or clipping. For the studies with decreased water availability relative to the ambient level (without decreased water availability), the treatment with decreased water availability was considered as the ‘control’ and ‘treatment’ using the ambient level. When more than one factor was manipulated in an experiment, we kept the other factors at the ambient level and then extracted the data on treatments for the focal factor.

### Bud bank type classification

**Figure 2:**
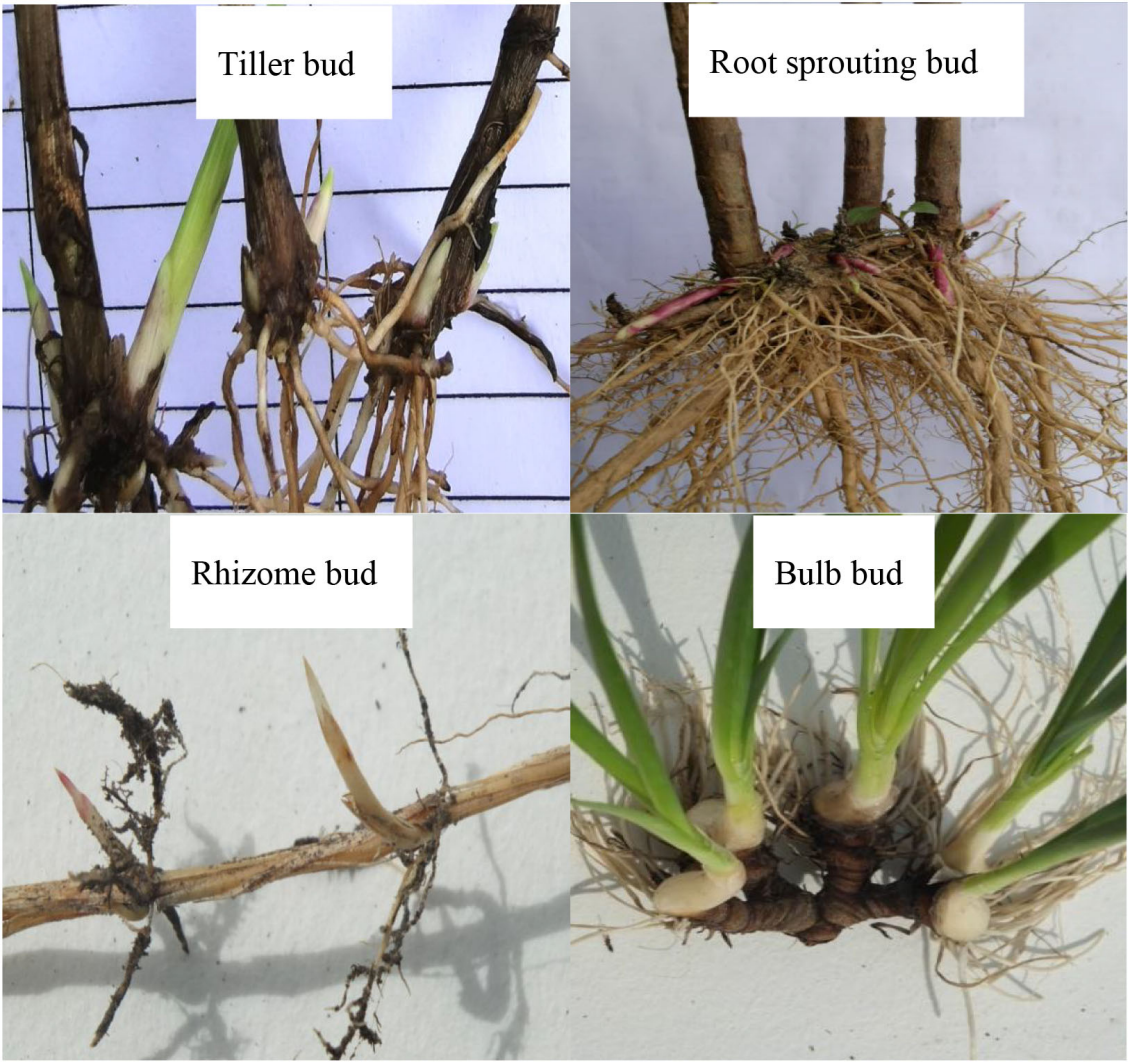
Belowground bud-bearing organ types covered in the analyzed dataset.

We classified the various bud banks based on their bud-bearing organs’ morphological characteristics. Thus, rhizome buds (axillary buds and apical buds on hypogeogenous rhizomes), tiller buds (axillary buds at the shoot bases of caespitose species and rhizomatous grasses), root-sprouting buds (adventitious buds formed mainly endogenously on roots of forb or shrub), and bulb buds, i.e., buds originating from the swollen bases of bulb-type species (see **Figure 2**). It is worth noting that buds on rhizomes and roots could be counted directly. In contrast, shoot bases need to be dissected for tiller bud counting.

According to papers or publications used in this meta-analysis, we classified bud into three viability classes, i.e., those that were metabolically active, dormant, or dead. Previous-year stem base halves were incubated in colorless triphenil tetrazolium chloride [TTC, 0.6% (w/v)] at 30°C in darkness for 15 h. Bud apexes that stained either red or pink were considered metabolically active. Change from colorless to either red or pink indicates an enzymatic reduction from TTC to insoluble red formazan. Buds unstained with TTC were tested using the vital stain Evan’s Blue [0.25% (w/v)], which does not penetrate intact semi-permeable membranes. Thus, unstained or dark blue-stained tissues using the vital stain were considered dormant or dead, respectively.

### The degree criteria for each moderator

We define the extent of the wildfire by the frequency (number of times). Less than 5 wildfires per year were considered low, less than 10 and more than 5 were considered moderate, and more than 10 were considered high. We defined the intensity of grazing according to the number of livestock per unit or the proportion of clipping, with less than 10 per hectare considered low, more than 10 and less than 30 considered moderate, and more than 30 considered high. A clipping ratio of less than 30% is considered low, between 30% and 50% is considered moderate, and greater than 50% is considered high. This definition follows the literature collected for the meta-analysis (see **Supplementary Table S2**).

To ensure consistency, we define the intensity of nitrogen addition by the amount and concentration added. Less than or equal to 20g per square meter was categorized as low, less than 20 mmol/L^-1^ as low, more than 20 mmol/L^-1^ but less than 40 mmol/L^-1^ as moderate, and more than 40 mmol/L^-1^ as high. We defined the intensity of drought according to the amount and proportion of rainfall intercepted as mentioned in literature. Less than 200 mm was categorized as low, greater than 200 mm and less than 500 mm was categorized as moderate, and greater than 500 mm was categorized as high. The proportion of intercepted rainfall less than 30% was considered low, greater than 30%, but less than 60% was considered moderate, and greater than 60% was considered high (see **Supplementary Table S2**).

### Effect size and variance computation

To examine the effects of N addition, drought, grazing, and wildfire disturbance on belowground bud bank density, we calculated the log response ratio (ln R) as the effect size of bud bank density for each component of N addition, drought, grazing, and wildfire disturbances for each study (Hedges et al., 1999):


$$\ln R=ln\left(\frac{{\bar{X}}_{t}}{{\bar{X}}_{c}}\right)=ln\left({\bar{X}}_{t}\right)-ln\left({\bar{X}}_{c}\right)$$

where ${\bar{X}}_{t}$ and ${\bar{X}}_{c}$ are the mean values of the individual bud bank density in the treatment (*t*) and control (*c*), respectively. The variance of ln R was calculated, following Hedges et al. (1999), as


$$v_{lnR}=\frac{{\left(SD_{c}\right)}^{2}}{N_{c}{\left({\bar{X}}_{c}\right)}^{2}}+\frac{{\left(SD_{t}\right)}^{2}}{N_{t}{\left({\bar{X}}_{t}\right)}^{2}}$$

where *N_c_* and *N_t_* are sample sizes, *SD_t_* and *SD_c_* are standard deviations, and *X_t_* and *X_c_* are mean values for the bud bank density in the treatment (*t*) and control (*c*), respectively. To avoid pseudo-replication, we pooled the multiple effect sizes (weighted by the inverse variance) and corresponding variances per study (Leimu et al., 2006). Pooling was done using the fixed-effect model (using the *rma* function in the R package METAFOR) because we assumed a single true underlying effect size in a study.

### Data analysis

All meta-analytical calculations and analyses were performed in R 3.1.3 (R Core Team, 2015) using the package METAFOR v1.9-7 (Viechtbauer, 2010). First, to test whether the bud bank densities of different plant functional types, on average, exhibited significant positive or negative responses to N addition, drought, grazing, and wildfire, we performed a general meta-analysis using a random-effect model (Gurevitch and Hedges, 2001). We computed weighted mean effect sizes and 95% confidence intervals (CIs) for each model for the moderator levels. We considered a mean effect size estimate significantly different from zero if the 95% CI around the mean did not include zero. For each of the disturbance, we compared mean effect sizes of different bud types (i.e., rhizome bud, tiller bud, root sprouting bud, and bulb bud; active bud and dormant bud) and plant functional type (i.e., forb, grass, sedge, shrub, and total plants). We also compared mean effect sizes of bud bank densities among different treatment levels (i.e., low, moderate, and high). In these models, total heterogeneity (QT) in effect sizes can be partitioned into heterogeneity explained by the model structure (QM) and unexplained heterogeneity (QE); we used the QT test (Koricheva et al., 2013) to test for a significant difference in the mean effect size among levels or groups for the moderator.

## Results

### Disturbance effects on belowground bud banks

**Figure 3:**
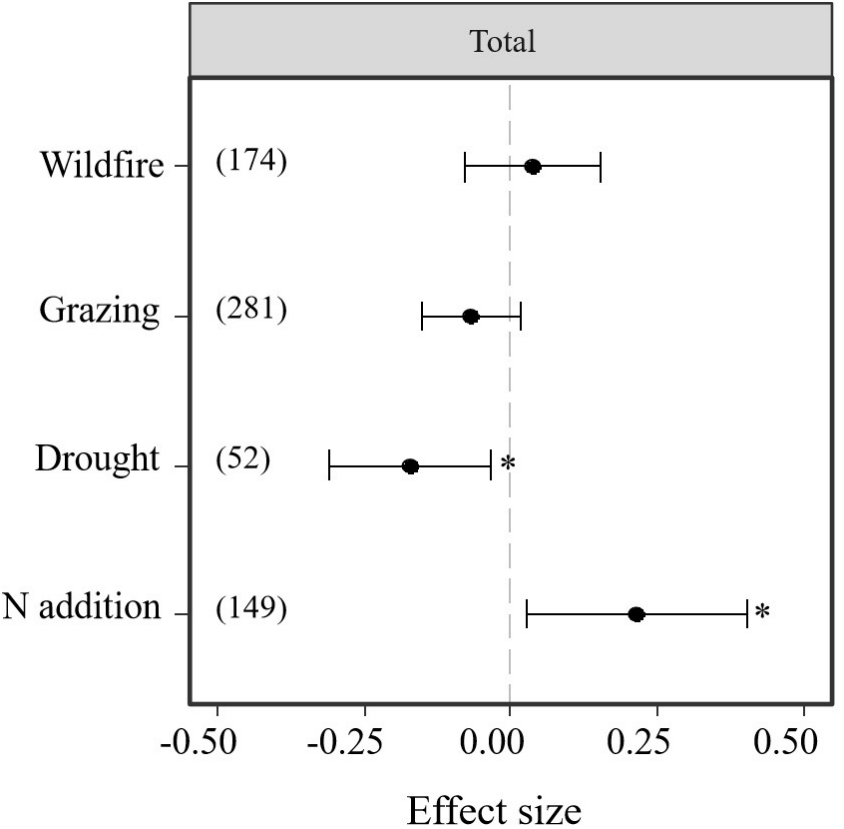
Responses (indicated by log response ratio mean effect sizes) of belowground bud bank densities to disturbances of wildfire, grazing, drought, and N addition. Error bars represent 95%-confidence intervals around the mean effect size estimates. The asterisk (*) indicates a statistically significant effect on the belowground bud bank density (i.e., *P < 0.05*), while ns denotes no significant effect. Sample sizes (i.e., the number of effect sizes) are given in parentheses. The dashed vertical line indicates zero effect of the global environmental change drivers.

The analysis of 48 studies indicated that the effect size of N addition and drought was higher than that of wildfire and grazing disturbances. Thus, drought and N addition significantly affected bud bank density but not wildfire and grazing (*P < 0.05*, **Table 1**; **Figure 3**). However, as drought imposed significantly negative effects on the belowground bud bank density (**Table 1**; **Figure 3**), N addition had significantly positive impacts on the belowground bud bank density (*P < 0.05*, **Table 1**; **Figure 3**). Neither wildfire nor grazing had significant effect on average belowground bud bank density (*P < 0.05*, **Table 1**; **Figure 3**).

**Table 1:** Results of meta-analysis comparing bud bank densities in responses to disturbances of wildfire, grazing, drought, and N addition.

Moderator	Number of effect sizes	Mean	Lower 95% CI	Upper 95% CI	P	Qtotal	Mean Study variance
Wildfire	174	0.0366	-0.076	0.1493	0.5297	838.827	0.3216
Grazing	281	-0.0687	-0.1538	0.0164	0.1137		
Drought	149	-0.1747	-0.3228	-0.0267	0.0207*	217.929	0.2911
N addition	52	0.2143	0.0117	0.4169	0.0382*		

The asterisk (*) indicates a statistically significant effect on the belowground bud bank density (*P* < 0.05).

### Effect of different degrees of disturbance on bud bank

Considering the level of the measured disturbance on bud banks, i.e., drought, N addition, fire, and grazing, we found that higher N addition and grazing levels negatively affected belowground bud bank densities. Both high-level and moderate-level of N addition significantly affected the belowground bud bank densities. However, moderate-level disturbances of drought, grazing, and fire had no significant effects on the belowground bud bank densities (*P < 0.05*, **Table 2**). Although both moderate and high-level of drought had no effect on the belowground bud bank density, surprisingly, low levels of drought had a minimal effect (i.e., indicated by a marginally significant effect) on it (*P < 0.1*, see **Table 2**).

**Table 2:** Results of meta-analysis comparing bud bank densities in responses to disturbances of wildfire, grazing, drought, and N addition.

Moderator	Level	Number of effect sizes	Mean	Lower 95% CI	Upper 95% CI	P	Qtotal	Mean Study Variance
Wildfire	Low	103	0.0205	-0.117	0.2181	0.5544	322.009	0.5798
	Moderate	4	0.5068	-0.3463	1.3598	0.2443		
	High	67	-0.0281	-0.2662	0.2101	0.8173		
Grazing	Low	65	0.0403	-0.0849	0.1655	0.5258	338.211	0.2009
	Moderate	169	-0.0533	-0.1369	0.0302	0.2108		
	High	47	-0.2306	-0.4043	-0.057	0.0092*		
Drought	Low	90	0.2620	-0.0275	0.5515	0.0761†	74.010	0.0868
	Moderate	14	0.2029	-0.4410	0.8468	0.5368		
	High	45	-0.0271	-0.6669	0.6126	0.9338		
N addition	Low	36	-0.0027	-0.1635	0.1581	0.9739	115.689	0.3812
	Moderate	8	-0.4199	-0.7596	-0.0802	0.0154*		
	High	8	-0.4257	-0.6800	-0.1715	0.0010*		

The asterisk (*) indicates a statistically significant effect on the belowground bud bank density (*i.e.*, *P* < 0.05), and † indicates a marginally significant effect on the belowground bud bank density (i.e., *P* < 0.1).

### Disturbance effects on different bud types

**Figure 4:**
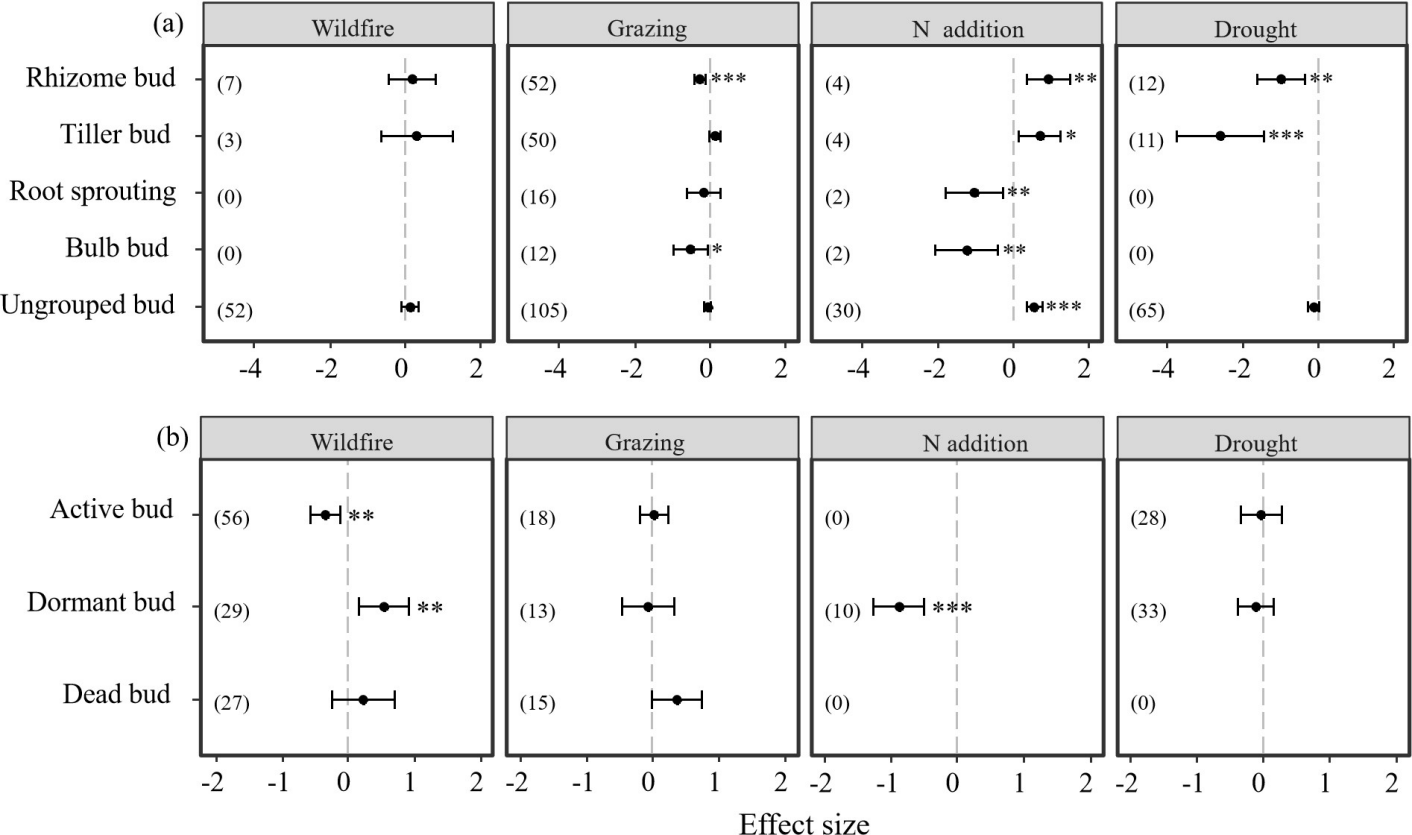
Responses of belowground bud bank densities of different bud types to disturbances of wildfire, grazing, drought, and N addition. Error bars represent 95%-confidence intervals around the mean effect size estimates. The asterisk (*, **, and ***) indicates a statistically significant effect on the belowground bud bank density (i.e., *P < 0.05*), while ns denotes no significant effect. Sample sizes (i.e., the number of effect sizes) are given in parentheses. The dashed vertical line indicates zero effect of the global environmental change drivers. **(A)** denotes the density of bud bank per unit area, **(B)** denotes the density of bud bank of per tiller, respectively.

Results also indicate that different bud types showed different responses to disturbances of N addition, drought, wildfire, and grazing (**Table 3**; **Figure 4**). Both rhizome buds and bulb buds showed significantly negative responses to grazing (*P < 0.05*, **Table 3**; **Figure 4A**). However, active and dormant bud showed significantly negative and positive responses, respectively, to wildfire (*P < 0.05*, **Table 3**; **Figure 4B**). Moreover, rhizome and tiller buds showed significantly positive responses to N addition, but the N addition imposed significantly negative effect on root sprouting, bulbs, and dormant buds (P < 0.05, **Table 3**; **Figure 4**). Additionally, the N addition had significantly positive effect on other buds that were not specifically grouped (i.e., ungrouped) in our dataset (*P < 0.05*, **Table 3**; **Figure 4**). Moreover, results showed that rhizome and tiller buds showed significantly negative responses to drought (*P < 0.05*, **Table 3**; **Figure 4A**). However, the drought had neither positive nor negative effect on the other buds (**Table 3**; **Figure 4**).

**Table 3:** Results of meta-analysis comparing bud bank densities to disturbances of wildfire, grazing, N addition, and drought.

Moderator	Group	Number of Effect sizes	Mean	Lower 95% CI	Upper 95% CI	P	Qtotal	Mean Study Variance
Wildfire	Rhizome bud	7	0.1853	-0.4496	0.8203	0.5673	326.952	0.5798
	Tiller bud	3	0.3098	-0.6314	1.2510	0.5189		
	Root sprouting bud	–	–	–	–	–		
	Buld bud	–	–	–	–	–		
	Ungrouped bud	52	0.1236	-0.1094	0.3566	0.2986		
	Active bud	56	-0.3164	-0.5429	-0.0899	0.0062*		
	Dormant bud	29	0.56	0.1849	0.9352	0.0034*		
	Dead bud	27	0.2544	-0.2209	0.7296	0.2942		
Grazing	Rhizome bud	52	-0.2599	-0.4084	-0.1113	0.0006*	501.094	0.1747
	Tiller bud	50	0.1292	-0.0170	0.2754	0.0834		
	Root sprouting bud	16	-0.1610	-0.5996	0.2776	0.4718		
	Buld bud	12	-0.5077	-0.9505	-0.0649	0.0246*		
	Ungrouped bud	105	-0.0568	-0.1509	0.0373	0.2369		
	Active bud	18	0.0253	-0.1905	0.241	0.8185		
	Dormant bud	13	-0.0685	-0.4583	0.3213	0.7304		
	Dead bud	15	0.3677	-0.0129	0.7462	0.0583		
N addition	Rhizome bud	4	0.9161	0.3413	1.4909	0.0018*	97.712	0.0868
	Tiller bud	4	0.6954	0.1366	1.2542	0.0147*		
	Root sprouting bud	2	-1.047	-1.8098	-0.2842	0.0071*		
	Buld bud	2	-1.2349	-2.0583	-0.4114	0.0033*		
	Ungrouped bud	30	0.5537	0.3519	0.7554	<0.0001*		
	Active bud	–	–	–	–	–		
	Dormant bud	10	-0.8779	-1.2609	-0.4948	<0.0001*		
	Dead bud	–	–	–	–	–		
Drought	Rhizome bud	12	-0.9829	-1.6122	-0.3536	0.0022*	152.364	0.3812
	Tiller bud	11	-2.5872	-3.7289	-1.4454	<0.0001*		
	Root sprouting bud	–	–	–	–	–		
	Buld bud	–	–	–	–	–		
	Ungrouped bud	65	-0.1162	-0.2703	0.038	0.1397		
	Active bud	28	-0.0339	-0.3421	0.2743	0.8292		
	Dormant bud	33	-0.1081	-0.3794	0.1632	0.4348		
	Dead bud	–	–	–	–	–		

The asterisk (*) indicates a statistically significant effect on the belowground bud bank density (*P* < 0.05). The asterisk (*) indicates a statistically significant effect on the belowground bud bank density (i.e., *P < 0.05*).

### Disturbance effects on plant functional type on bud banks

**Figure 5:**
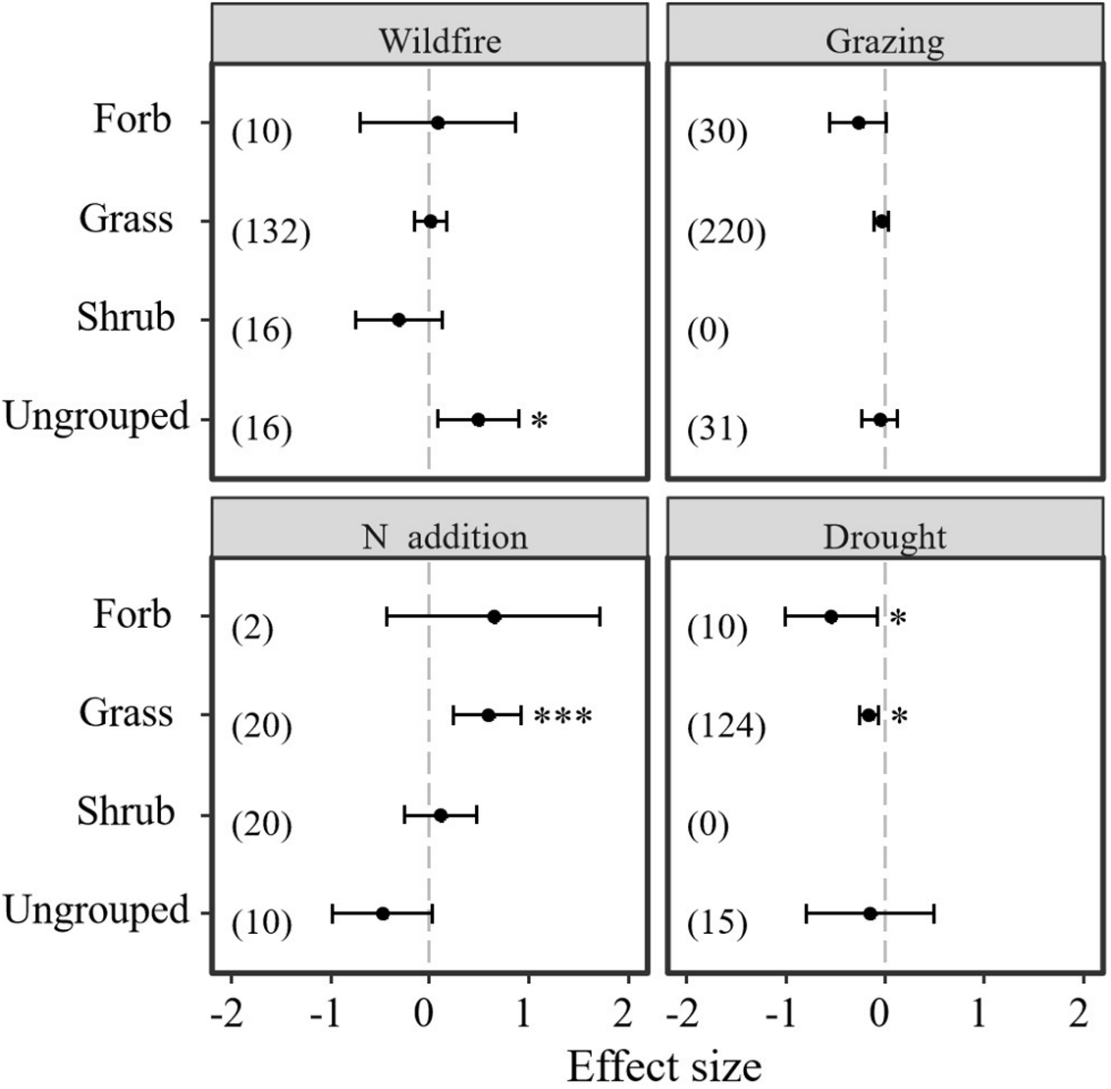
Responses of belowground bud bank densities of different plant functional groups to disturbances of wildfire, grazing, drought, and N addition. Error bars represent 95%-confidence intervals around the mean effect size estimates. The asterisk (*) indicates a statistically significant effect on the belowground bud bank density (P < 0.05), The asterisk (***) indicates a statistically significant effect on the belowground bud bank density (P < 0.001), respectively, while ns denotes no significant effect. Sample sizes (i.e., the number of effect sizes) are given in parentheses. The dashed vertical line indicates zero effect of the global environmental change drivers.

Results indicate that different plant functional types showed different responses to the disturbances of N addition, drought, wildfire, and grazing (**Table 4**; **Figure 5**). Neither wildfire nor grazing had a significant positive or negative effect on plant functional type (grasses, forbs, and shrubs) (**Table 4**; **Figure 5**). However, ungrouped plant functional types showed significantly positive response to wildfire (**Table 4**; **Figure 5**). Unlike wildfire and grazing, N addition and drought significantly affected grasses and forbs but not shrubs. Thus, grasses and forbs showed significantly negative responses to drought. However, N addition significantly promoted the bud bank density of grasses (**Table 4**; **Figure 5**).

**Table 4:** Results of meta-analysis comparing bud bank densities of different plant functional groups to disturbances of wildfire, grazing, N addition, and drought.

Moderator	Group	Number of Effect sizes	Mean	Lower 95% CI	Upper 95% CI	P	Qtotal	Mean Study Variance
Wildfire	Forb	10	0.0814	-0.7041	0.8669	0.8391	326.952	0.5798
	Grass	132	0.0134	-0.1477	0.1746	0.8702		
	Shrub	16	-0.3177	-0.7575	0.122	0.1567		
	Ungrouped	16	0.491	0.0833	0.8987	0.0182*		
Grazing	Forb	30	-0.2756	-0.5636	0.0123	0.0606	501.094	0.1747
	Grass	220	-0.0396	-0.1126	0.0334	0.2876		
	Shrub	–	–	–	–	–		
	Ungrouped	31	-0.0534	-0.2363	0.1296	0.5675		
N addition	Forb	2	0.6444	-0.432	1.7208	0.2406	97.712	0.0868
	Grass	20	0.5893	0.2462	0.9324	0.0008*		
	Shrub	20	0.1096	-0.2569	0.4761	0.5577		
	Ungrouped	10	-0.4807	-0.9784	0.0171	0.0584†		
Drought	Forb	10	-0.5414	-1.0095	-0.0734	0.0430*	152.364	0.3812
	Grass	124	-0.1603	-0.2574	-0.0632	0.0492*		
	Shrub	–	–	–	–	–		
	Ungrouped	15	-0.1555	-0.8006	0.4896	0.6365		

The asterisk (*) indicates a statistically significant effect on the belowground bud bank density (*P* < 0.05). The asterisk (*) indicates a statistically significant effect on the belowground bud bank density (i.e., *P* < 0.05).

## Discussion

Results of this meta-analysis provide empirical evidence that N addition and drought impose significantly divergent effects, which depend on the degree and frequency of disturbances, plant functional types, and bud types. Overall, our results confirmed the findings of most previous studies reporting that drought (Adomako et al., 2020b; Buttler et al., 2019; Lei et al., 2020; Xu et al., 2021) and N addition (Gough et al., 2012; Ren et al., 2019, 2023; Zheng et al., 2019) affect plant growth and productivity.

Drought events hamper the regeneration of belowground bud banks (Qian et al., 2023; Wang et al., 2019), profoundly affecting ecosystem succession and community composition and dynamics (Buttler et al., 2019; Lei et al., 2020; Qian et al., 2022; Wang et al., 2019). In the present analysis, drought significantly impacts forbs and grasses, key plant functional types in many grassland ecosystems worldwide (Petermann and Buzhdygan, 2021; Qian et al., 2023; Wang et al., 2019). This observation could underlie the significant losses and degradation of global grassland ecosystems, particularly in temperate grasslands of Asia and North America and tropical grasslands of South America and Africa. For instance, about 99% of North America’s aboveground regrowth of tallgrass ecosystems is recruited from the belowground bud banks (Benson and Hartnett, 2006). Moreover, a reduction of 66% of annual precipitation, indicating a severe drought, significantly reduced the bud density, consequently impacting the community’s aboveground shoot productivity (Qian et al., 2023). Although in a short term, a 90-day drought significantly reduced the net productivity (the sum of aboveground and belowground biomass) of rhizomatous grass *Leymus chinensis* by 69% and decreased belowground bud bank density by 56% (Wang et al., 2019). This suggests that *L. chinensis*, which is native to China and is dominant in the Eurasian steppe ecosystems, can easily be lost due to prolonged drought regimes (Adomako et al., 2021; Wang et al., 2019). Our results suggest that drought is critical to grassland ecosystems owing to its immediate reduction effects on net primary productivity and future productivity of grassland due to its impacts on belowground bud bank density.

However, effects of the drought largely depend on its magnitude or severity, coupled with plant functional and bud types. For example, in a grassland experimental community in Central Texas, severe drought profoundly reduced biomass productivity by 82% (Xu et al., 2017). Such negative effects on grasses were primarily attributed to reduced growth, tiller number, and rhizome buds (Zhuang et al., 2017), as well as ramets, root sprouting, and dormant buds for forbs (Saud et al., 2017). Previous studies have consistently reported that bud bank density and bud types of forbs showed the highest vulnerability to drought compared to grasses (Li et al., 2021; Qian et al., 2021, 2023). These differential responses may explain the disparities in ecosystem-level responses to the ongoing environmental disturbance (Bobbink et al., 2010; Stevens et al., 2004). Thus, ecosystems in which grasses are key dominant species may show stronger resistance than where forbs dominate (Li et al., 2016; You et al., 2017). However, in a long-term drought event, how bud banks of grasses may respond and their impact on the wider grassland vegetation require further experimental clarification.

Furthermore, results suggest that N addition significantly promoted bud bank density and aboveground growth (Qian et al., 2021). One key mechanism underpinning such observations is that N addition enhances litter quality and accumulation, promoting soil physico-chemical characteristics and their positive feedback on belowground bud banks (Hou et al., 2021; Wu et al., 2024). N enrichment and litter addition jointly enhanced bud numbers and aboveground growth, suggesting that N addition may be tightly linked with ecosystem-level growth and productivity (Li et al., 2021; Ren et al., 2024). However, N addition mostly exhibits positive effects at the plant species level under short-term conditions but cascades profound negative effects on population- and community-level diversity and dynamics (Adomako et al., 2020a; Gao et al., 2022; Li et al., 2022). Thus, increasing N addition promotes large canopy formation of species (e.g., grasses) with high nutrient use efficiency, decreasing light entry and water availability to understory plants (Wu and Yu, 2022; Xing et al., 2022), as well as decreasing soil temperature and respiration (Li et al., 2018; Yang et al., 2022). Eventually, such species dominate their ecosystems by competitively excluding weaker species (Gough et al., 2012; Liu et al., 2021). This phenomenon partly explains the loss of many species by elevated N deposition under the ongoing global environmental change (Li et al., 2022; Ren et al., 2022; Stevens et al., 2010). For example, earlier studies have attributed severe N deposition as driving the loss of species richness of grasslands across Europe and Great Britain (Stevens et al., 2004, 2010).

Indeed, N addition effects on belowground bud banks and aboveground growth recruitments strongly correlate with the bud type and degree of N availability, as observed from our analysis. Previous studies have demonstrated that high N addition promotes vegetative growth, including rhizomes, tillers, and ramets (Adomako et al., 2022; Gough et al., 2012). Also, it has been previously indicated that rhizomatous buds are highly vulnerable to nutrient shortages and intense drought in grassland ecosystems (Qian et al., 2017). For example, a recent long- and short-term grassland study designed to test the N addition duration on clumper, stoloniferous, and rhizomatous clonal growth forms found that short-term N addition promoted the growth of the clumper clonal growth form (Zheng et al., 2019). The authors, however, reported that long-term N addition significantly suppressed stoloniferous clonal growth but remarkably favored rhizomatous clonal growth. A recent meta-analysis and some studies found that grasses and forbs responded differently to N addition (You et al., 2017). There has been a general recognition that N addition promotes the aboveground and belowground biomass of grasses but reduces that of forbs (Adomako and Yu, 2023; Cheng et al., 2023; Song et al., 2011; You et al., 2017). Our results and previous findings suggest that drought and N addition effects on bud banks and bud types depend on the magnitude of disturbances and specific plant functional type.

Although wildfire and grazing did not affect belowground bud bank densities, high grazing intensity significantly impacts bud types, particularly active, dormant, rhizome, and bulb buds. Such bud-type-specific effects can undermine ecosystems where these bud types dominate the belowground structures. In the Eurasian regions where these species dominate the ecosystem, the loss of below- and above-ground biodiversity caused by extreme grazing has resulted in grassland degradation (Liang et al., 2021). Perhaps overgrazing is among the most important disturbances driving the loss of grassland biomes worldwide (Liang et al., 2021; Osem et al., 2002; Ren et al., 2018; Zhang et al., 2023), resulting from its multifaceted damaging effects on below- and above-ground biodiversity (Cao et al., 2024; Liang et al., 2021), soil respiration and organic carbon (Li et al., 2024), and ecosystem multifunctionality (Zhang et al., 2023).

Similarly, while wildfire did not impact bud bank densities, it significantly induced negative and positive impacts on active and dormant buds. The majority of studies have documented drastic effects of wildfire on belowground processes (Clarke et al., 2022; Kong et al., 2022), nutrient availability (Kong et al., 2022), and plant aboveground productivity (Roces-Díaz et al., 2022; Wardle et al., 2003), all of which positively correlate with regeneration of active buds in ecosystems. Surprisingly, wildfire exerted promotional effects on dormant buds in the present analysis, which is consistent with few previous studies that have documented positive impacts of wildfire on grassland regrowth, especially in the semi-arid regions of Texas, USA (Fultz et al., 2016) and as management strategy of protected forest in Northeast Portugal (Fonseca et al., 2011). Our analysis indicated that N addition and drought would differentially impact specific attributes of belowground bud banks, such as plant functional types and bud types.

## Conclusions

Results of our meta-analysis suggest that N addition and drought significantly impact bud bank density, potentially affecting plant populations, community-level productivity, and ecosystem stability. This analysis confirms many predictions of N deposition effects on global ecosystems in the coming decades. Results consistently replicated most previous findings, which suggest that drought adversely affects belowground bud bank densities and numbers with cascading consequences for aboveground productivity. Moreover, N addition significantly promotes belowground bud bank density, positively correlating with aboveground biomass, litter accumulation, and subsequently increased nutrient availability. The disparity effects on belowground bud banks among the measured variables may be attributed to the dependency of some factors (e.g., wildfire) on drought variables. Thus, for example, effects of wildfire on grassland ecosystems increase with drought intensity and duration. Given the importance of grassland ecosystems and the predicted increases in N deposition and drought in the coming decades, prioritizing the management of belowground bud banks will remain a critical component of maintaining the productivity and stability of grassland globally.

## Data Availability

The raw data supporting the conclusions of this article will be made available by the authors, without undue reservation.

## References

[B1] Adomako M. O. Gao F.-L. Li J.-M. Du D.-L. Xue W. Yu F.-H. (2020a). Effects of soil nutrient heterogeneity and parasitic plant infection on an experimental grassland community. Flora 271, 151666. doi: 10.1016/j.flora.2020.151666

[B2] Adomako M. O. Xue W. Du D.-L. Yu F.-H. (2021). Soil biota and soil substrates influence responses of the rhizomatous clonal grass *Leymus chinensis* to nutrient heterogeneity. Plant Soil 465, 19–29. doi: 10.1007/s11104-021-04967-0

[B3] Adomako M. O. Xue W. Du D.-L. Yu F.-H. (2022). Soil microbe-mediated N:P stoichiometric effects on *Solidago canadensis* performance depend on nutrient levels. Microb. Ecol. 83, 960–970. doi: 10.1007/s00248-021-01814-8 34279696

[B4] Adomako M. O. Xue W. Tang M. Du D.-L. Yu F.-H. (2020b). Synergistic effects of soil microbes on *Solidago canadensis* depend on water and nutrient availability. Microb. Ecol. 80, 837–845. doi: 10.1007/s00248-020-01537-2 32561944

[B5] Adomako M. O. Yu F.-H. (2023). Functional group dominance-mediated N:P effects on community productivity depend on soil nutrient levels. Rhizosphere 26, 100692. doi: 10.1016/j.rhisph.2023.100692

[B6] Bardgett R. D. Bullock J. M. Lavorel S. Manning P. Schaffner U. Ostle N. . (2021). Combatting global grassland degradation. Nat. Rev. Earth Env. 2, 720–735. doi: 10.1038/s43017-021-00207-2

[B7] Benson E. Hartnett D. (2006). The role of seed and vegetative reproduction in plant recruitment and demography in tallgrass prairie. Plant Ecol. 187, 163–178. doi: 10.1007/s11258-005-0975-y

[B8] Bobbink R. Hicks K. Galloway J. Spranger T. Alkemade R. Ashmore M. . (2010). Global assessment of nitrogen deposition effects on terrestrial plant diversity: a synthesis. Ecol. Appl. 20, 30–59. doi: 10.1890/08-1140.1 20349829

[B9] Buttler A. Mariotte P. Meisser M. Guillaume T. Signarbieux C. Vitra A. . (2019). Drought-induced decline of productivity in the dominant grassland species Lolium perenne L. depends on soil type and prevailing climatic conditions. Soil Bio. Biochem. 132, 47–57. doi: 10.1016/j.soilbio.2019.01.026

[B10] Cao F. Li W. Jiang Y. Gan X. Zhao C. Ma J. (2024). Effects of grazing on grassland biomass and biodiversity: A global synthesis. Field Crop Res. 306, 109204. doi: 10.1016/j.fcr.2023.109204

[B11] Carter D. L. VanderWeide B. L. Blair J. M. (2012). Drought-mediated stem and below-ground bud dynamics in restored grasslands. Appl. Veg. Sci. 15, 470–478. doi: 10.1111/j.1654-109x.2012.01200.x

[B12] Cheng Y. Liu X. Song Z. Ma M. Zhou S. Allan E. (2023). Divergent trait responses to nitrogen addition in tall and short species. J. Ecol. 111, 1443–1454. doi: 10.1111/1365-2745.14108/v1/review1

[B13] Chikamoto Y. Timmermann A. Widlansky M. J. Balmaseda M. A. Stott L. (2017). Multi-year predictability of climate, drought, and wildfire in southwestern North America. Sci. Rep. 7, 6568. doi: 10.1038/s41598-017-06869-7 28747719 PMC5529505

[B14] Ciais P. Reichstein M. Viovy N. Granier A. Ogée J. Allard V. . (2005). Europe-wide reduction in primary productivity caused by the heat and drought in 2003. Nature 437, 529–533. doi: 10.1038/nature03972 16177786

[B15] Clarke H. Nolan R. H. De Dios V. R. Bradstock R. Griebel A. Khanal S. . (2022). Forest fire threatens global carbon sinks and population centres under rising atmospheric water demand. Nat. Commun. 13, 7161. doi: 10.1038/s41467-022-34966-3 36418312 PMC9684135

[B16] Dalgleish H. J. Hartnett D. C. (2009). The effects of fire frequency and grazing on tallgrass prairie productivity and plant composition are mediated through bud bank demography. Plant Ecol. 201, 411–420. doi: 10.1007/978-90-481-2798-6_4

[B17] Dawson T. P. Jackson S. T. House J. I. Prentice I. C. Mace G. M. (2011). Beyond predictions: biodiversity conservation in a changing climate. Science 332, 53–58. doi: 10.1126/science.1200303 21454781

[B18] Debinski D. M. Wickham H. Kindscher K. Caruthers J. C. Germino M. (2010). Montane meadow change during drought varies with background hydrologic regime and plant functional group. Ecology 91, 1672–1681. doi: 10.1890/09-0567.1 20583709

[B19] Donovan V. M. Twidwell D. Uden D. R. Tadessem T. Wardlowm B. D. Bielskim C. H. . (2020). Resilience to large, “Catastrophic” Wildfires in north america’s grassland biome. Earth’s Future 8, e2020EF001487. doi: 10.1029/2020ef001487

[B20] Fischer E. M. Knutti R. (2014). Detection of spatially aggregated changes in temperature and precipitation extremes. Geophys. Res. Lett. 41, 547–554. doi: 10.1002/2013gl058499

[B21] Fonseca F. Leite M. M. Figueiredo T. D. (2011). “Soil properties in burned and unburned Mediterranean shrublands of Montesinho Natural Park, Northeast Portugal,” in Proceedings of the 3rd International Meeting of Fire Effects on Soil Properties (Universidade do Minho).

[B22] Fultz L. M. Moore-Kucera J. Dathe J. Davinic M. Perry G. Wester D. . (2016). Forest wildfire and grassland prescribed fire effects on soil biogeochemical processes and microbial communities: Two case studies in the semi-arid Southwest. Appl. Soil Ecol. 99, 118–128. doi: 10.1002/2013gl058499

[B23] Gao S. Song Y. Song C. Wang X. Gong C. Ma X. . (2022). Long-term nitrogen addition alters peatland plant community structure and nutrient resorption efficiency. Sci. Total Environ. 844, 157176. doi: 10.2139/ssrn.4107535 35803431

[B24] Gough L. Gross K. L. Cleland E. E. Clark C. M. Collins S. L. Fargione J. E. . (2012). Incorporating clonal growth form clarifies the role of plant height in response to nitrogen addition. Oecologia 169, 1053–1062. doi: 10.1007/s00442-012-2264-5 22302512

[B25] Gurevitch J. Hedges L. V. (2001). “Meta-analysis: combining the results of independent experiments,” in Design and analysis of ecological experiments. Eds. Scheiner S. M. Gurevitch J. (Oxford, UK: Oxford University Press), 347–369.

[B26] Hedges L. V. Gurevitch J. Curtis P. S. (1999). The meta-analysis of response ratios in experimental ecology. Ecology 80, 1150–1156. doi: 10.2307/177062

[B27] Hoover D. L. Knapp A. K. Smith M. D. (2014). Resistance and resilience of a grassland ecosystem to climate extremes. Ecology 95, 2646–2656. doi: 10.1890/13-2186.1

[B28] Hou S.-L. Hättenschwiler S. Yang J.-J. Sistla S. Wei H.-W. Zhang Z.-W. . (2021). Increasing rates of long-term nitrogen deposition consistently increased litter decomposition in a semi-arid grassland. New Phytol. 229, 296–307. doi: 10.1111/nph.16854 32762047

[B29] Klimešová J. Klimeš L. (2007). Bud banks and their role in vegetative regeneration – A literature review and proposal for simple classification and assessment. Perspect. Plant Ecol. 8, 115–129. doi: 10.1016/j.ppees.2006.10.002

[B30] Klimešová J. Martínková J. Bartušková A. Ott J. P. (2023). Belowground plant traits and their ecosystem functions along aridity gradients in grasslands. Plant Soil 1, 39–48. doi: 10.1007/s11104-023-05964-1

[B31] Klimešová J. Ottaviani G. Charles-Dominique T. Campetella G. Canullo R. Chelli S. . (2021). Incorporating clonality into the plant ecology research agenda. Trends Plant Sci. 26, 1236–1247. doi: 10.1016/j.tplants.2021.07.019 34419339

[B32] Kong J. Xiang X. Yang J. (2022). Wildfire alters the linkage between total and available soil C:N:P ratios and the stoichiometric effects on fine root growth in a Chinese boreal larch forest. Plant Soil 471, 211–225. doi: 10.1007/s11104-021-05215-1

[B33] Koricheva J. Gurevitch J. Mengersen K. (eds.). (2013). Handbook of meta-analysis in ecology and evolution (Princeton, NJ: Princeton University Press).

[B34] Lei T. Feng J. Lv J. Wang J. Song H. Song W. . (2020). Net primary productivity loss under different drought levels in different grassland ecosystems. J. Environ. Manage. 274, 111144. doi: 10.1016/j.jenvman.2020.111144 32798851

[B35] Leimu R. Mutikainen P. I. A. Koricheva J. Fischer M. (2006). How general are positive relationships between plant population size, fitness and genetic variation. J. Ecol. 94, 942–952. doi: 10.1111/j.1365-2745.2006.01150.x

[B36] Leys B. A. Marlon J. R. Umbanhowar C. Vannière B. (2018). Global fire history of grassland biomes. Ecol. Evol. 8, 8831–8852. doi: 10.1002/ece3.4394 30271549 PMC6157676

[B37] Li C. Peng Y. Nie X. Yang Y. Yang L. Li F. . (2018). Differential responses of heterotrophic and autotrophic respiration to nitrogen addition and precipitation changes in a Tibetan alpine steppe. Sci. Rep. 8, 16546. doi: 10.1038/s41598-018-34969-5 30410000 PMC6224420

[B38] Li J. H. Zhang J. Li W. J. Xu D. H. Knops J. M. H. Du G. Z. (2016). Plant functional groups, grasses versus forbs, differ in their impact on soil carbon dynamics with nitrogen fertilization. Eur. J. Soil Biol. 75, 79–87. doi: 10.1016/j.ejsobi.2016.03.011

[B39] Li S. Xing T. Sa R. Zhang Y. Chen H. Jin K. . (2024). Effects of grazing on soil respiration in global grassland ecosystems. Soil Till. Res. 238, 106033. doi: 10.1016/j.still.2024.106033

[B40] Li W. Luo S. Wang J. Zheng X. Zhou X. Xiang Z. . (2022). Nitrogen deposition magnifies destabilizing effects of plant functional group loss. Sci. Total Environ. 835, 155419. doi: 10.1016/j.scitotenv.2022.155419 35483460

[B41] Li Z. Wu J. Han Q. Nie K. Xie J. Li Y. . (2021). Nitrogen and litter addition decreased sexual reproduction and increased clonal propagation in grasslands. Oecologia 195, 131–144. doi: 10.1007/s00442-020-04812-8 33491109

[B42] Liang M. Liang C. Hautier Y. Wilcox K. R. Wang S. (2021). Grazing-induced biodiversity loss impairs grassland ecosystem stability at multiple scales. Ecol. Lett. 24, 2054–2064. doi: 10.1111/ele.13826/v2/review2 34319652

[B43] Liu L. Cheng J. Li Y. Lan Z. Bai Y. (2021). N-enrichment induced biodiversity loss can be explained by reductions in competitive intransitivity: Evidence from a decade-long grassland experiment. Environ. Exp. Bot. 184, 104372. doi: 10.1016/j.envexpbot.2021.104372

[B44] Luo W. Muraina T. O. Griffin-Nolan R. J. Ma W. Song L. Fu W. . (2023). Responses of a semiarid grassland to recurrent drought are linked to community functional composition. Ecology 104, e3920. doi: 10.1002/ecy.3920 36416074

[B45] Mackie K. A. Zeiter M. Bloor J. M. G. Stampfli A. (2019). Plant functional groups mediate drought resistance and recovery in a multisite grassland experiment. J. Ecol. 107, 937–949. doi: 10.1111/1365-2745.13102

[B46] Meng B. Li J. Maurer G. E. Zhong S. Yao Y. Yang X. . (2021). Nitrogen addition amplifies the nonlinear drought response of grassland productivity to extended growing-season droughts. Ecology 102, e03483. doi: 10.1002/ecy.3483 34287849

[B47] Osem Y. Perevolotsky A. Kigel J. (2002). Grazing effect on diversity of annual plant communities in a semi-arid rangeland: interactions with small-scale spatial and temporal variation in primary productivity. J. Ecol. 90, 936–946. doi: 10.1046/j.1365-2745.2002.00730.x

[B48] Ott J. P. Klimešová J. Hartnett D. C. (2019). The ecology and significance of below-ground bud banks in plants. Ann. Bot. 123, 1099–1118. doi: 10.1093/aob/mcz051 31167028 PMC6612937

[B49] Pecl G. T. Araújo M. B. Bell J. D. Blanchard J. Bonebrake T. C. Chen I. C. . (2017). Biodiversity redistribution under climate change: Impacts on ecosystems and human well-being. Science 355, eaai9214. doi: 10.1126/science.aai9214 28360268

[B50] Petermann J. S. Buzhdygan O. Y. (2021). Grassland biodiversity. Curr. Biol. 31, R1195–R1201. doi: 10.1016/j.cub.2021.06.060 34637731

[B51] Pontes-Lopes A. Silva C. V. J. Barlow J. Rincón L. M. Campanharo W. A. Nunes C. A. . (2021). Drought-driven wildfire impacts on structure and dynamics in a wet Central Amazonian forest. P. R. Soc b-Biol. Sci. 288, 20210094. doi: 10.1098/rspb.2021.0094 PMC8131120 34004131

[B52] Qian J. Guo Z. Muraina T. O. Te N. Griffin-Nolan R. J. Song L. . (2022). Legacy effects of a multi-year extreme drought on belowground bud banks in rhizomatous vs bunchgrass-dominated grasslands. Oecologia 198, 763–771. doi: 10.1007/s00442-022-05133-8 35230515

[B53] Qian J. Wang Z. Klimešová J. Lü X. Zhang C. (2021). Belowground bud bank and its relationship with aboveground vegetation under watering and nitrogen addition in temperate semiarid steppe. Ecol. Indic. 125, 107520. doi: 10.1016/j.ecolind.2021.107520

[B54] Qian J. Wang Z. Liu Z. Busso C. A. (2017). Belowground bud bank responses to grazing intensity in the Inner-Mongolia Steppe, China. Land Degrad. Dev. 28, 822–832. doi: 10.1002/ldr.2300

[B55] Qian J. Zhang Z. Dong Y. Ma Q. Yu Q. Zhu J. . (2023). Responses of bud banks and shoot density to experimental drought along an aridity gradient in temperate grasslands. Funct. Ecol. 37, 1211–1220. doi: 10.1111/1365-2435.14301

[B56] R Core Team . (2015) R: a language and environment for statistical computing (Vienna, Austria: R Foundation for Statistical Computing). Available at: http://www.R-project.org/ (Accessed October 1, 2015).

[B57] Rasband W. S. (2013) (Bethesda, Maryland, USA: U.S. National Institutes of Health). Available at: http://imagej.nih.gov/ij/ (Accessed October 1, 2015).

[B58] Ren G.-Q. Cui M. Yu H. Fan X. Zhu Z. Zhang H. . (2024). Global environmental change shifts ecological stoichiometry coupling between plant and soil in early-stage invasions. J. Soil Sci. Plant Nut 24, 2402–2412. doi: 10.1007/s42729-024-01659-3

[B59] Ren G.-Q. Du Y. Yang B. Wang J. Cui M. Dai Z. . (2023). Influence of precipitation dynamics on plant invasions: response of alligator weed (Alternanthera philoxeroides) and co-occurring native species to varying water availability across plant communities. Biol. Invasions 25, 519–532. doi: 10.1007/s10530-022-02931-2

[B60] Ren G.-Q. Li Q. Li Y. Li J. Adomako M. O. Dai Z.-C. . (2019). The enhancement of root biomass increases the competitiveness of an invasive plant against a co-occurring native plant under elevated nitrogen deposition. Flora 261, 151486. doi: 10.1016/j.flora.2019.151486

[B61] Ren G.-Q. Yang B. Cui M. Dai Z. Xiang Y. Zhang H. . (2022). Warming and elevated nitrogen deposition accelerate the invasion process of Solidago canadensis L. Ecol.Process. 11, 62. doi: 10.1186/s13717-022-00407-8

[B62] Ren H. Eviner V. T. Gui W. Wilson G. W. T. Cobb A. B. Yang G. . (2018). Livestock grazing regulates ecosystem multifunctionality in semi-arid grassland. Func. Ecol. 32, 2790–2800. doi: 10.1111/1365-2435.13215

[B63] Roces-Díaz J. V. Santín C. Martínez-Vilalta J. Doerr S. H. (2022). A global synthesis of fire effects on ecosystem services of forests and woodlands. Front. Ecol. Environ. 20, 170–178. doi: 10.1002/fee.2349

[B64] Ru J. Wan S. Hui D. Song J. (2023). Overcompensation of ecosystem productivity following sustained extreme drought in a semiarid grassland. Ecology 104, e3997. doi: 10.1002/ecy.3997 36799428

[B65] Saud S. Fahad S. Yajun C. Ihsan M. Z. Hammad H. M. Nasim W. . (2017). Effects of nitrogen supply on water stress and recovery mechanisms in Kentucky bluegrass plants. Fronti. Plant Sci. 8. doi: 10.3389/fpls.2017.00983 PMC5463276 28642781

[B66] Schulte to Bühne H. Tobias J. A. Durant S. M. Pettorelli N. (2021). Improving predictions of climate change–land use change interactions. Trends Ecol. Evol. 36, 29–38. doi: 10.1016/j.tree.2020.08.019 33020018

[B67] Song L. Bao X. Liu X. Zhang Y. Christie P. Fangmeier A. . (2011). Nitrogen enrichment enhances the dominance of grasses over forbs in a temperate steppe ecosystem. Biogeosciences 8, 2341–2350. doi: 10.5194/bg-8-2341-2011

[B68] Stevens C. J. Dise N. B. Mountford J. O. Gowing D. J. (2004). Impact of nitrogen deposition on the species richness of grasslands. Science 303, 1876–1879. doi: 10.1126/science.1094678 15031507

[B69] Stevens C. J. Duprè C. Dorland E. Gaudnik C. Gowing D. J. G. Bleeker A. . (2010). Nitrogen deposition threatens species richness of grasslands across Europe. Environ. pollut. 158, 2940–2945. doi: 10.1016/j.envpol.2010.06.006 20598409

[B70] Tonkin J. D. Bogan M. T. Bonada N. Rios-Touma B. Lytle D. A. (2017). Seasonality and predictability shape temporal species diversity. Ecology 98, 1201–1216. doi: 10.1002/ecy.1761 28144975

[B71] Twidwell D. Rogers W. E. Wonkka C. L. Taylor C. A. Kreuter U. P. (2016). Extreme prescribed fire during drought reduces survival and density of woody resprouters. J. Appl. Ecol. 53, 1585–1596. doi: 10.1111/1365-2664.12674

[B72] Viechtbauer W. (2010). Conducting meta-analyses in R with the metafor package. J. Stat. Softw 36, 1–48. doi: 10.18637/jss.v036.i03

[B73] Wang J. Shi Y. Ao Y. Yu D. Wang J. Gao S. . (2019). Summer drought decreases *Leymus chinensis* productivity through constraining the bud, tiller and shoot production. J. Agron. Crop Sci. 205, 554–561. doi: 10.1111/jac.12354

[B74] Wang S. Duan J. Xu G. Wang Y. Zhang Z. Rui Y. . (2012). Effects of warming and grazing on soil N availability, species composition, and ANPP in an alpine meadow. Ecology 93, 2365–2376. doi: 10.1890/11-1408.1 23236908

[B75] Wardle D. A. Hörnberg G. Zackrisson O. Kalela-Brundin M. Coomes D. A. (2003). Long-term effects of wildfire on ecosystem properties across an Island area gradient. Science 300, 972–975. doi: 10.1126/science.1082709 12738863

[B76] White E. R. Hastings A. (2020). Seasonality in ecology: Progress and prospects in theory. Ecol. Complex. 44, 100867. doi: 10.7287/peerj.preprints.27235v1

[B77] Wragg P. D. Mielke T. Tilman D. (2018). Forbs, grasses, and grassland fire behaviour. J. Ecol. 106, 1983–2001. doi: 10.1111/1365-2745.12980

[B78] Wu J. Hou X. Xu L. Zhou Q. Wang Y. Guo Z. . (2024). Belowground bud banks and land use change: Rolesof vegetation and soil properties in mediating the composition of bud banks in different ecosystems. Front. Plant Sci. 14. doi: 10.3389/fpls.2023.1330664 PMC10796614 38250452

[B79] Wu J. Yu F.-H. (2022). Belowground bud bank of invasive plants contributes to their successful invasion in coastal wetlands. Restor. Ecol. 31, e13821. doi: 10.1111/rec.13821

[B80] Xing A. Xu L. Zhao M. Shen H. Ma S. Fang J. (2022). Shifts in understory plant composition induced by nitrogen addition predict soil fungal beta diversity in a boreal forest. Biol. Fert. Soils 58, 667–677. doi: 10.1007/s00374-022-01652-x

[B81] Xu C. Ke Y. Zhou W. Luo W. Ma W. Song L. . (2021). Resistance and resilience of a semi-arid grassland to multi-year extreme drought. Ecol. Indic. 131, 108139. doi: 10.1016/j.ecolind.2021.108139

[B82] Xu X. Polley H. W. Hofmockel K. Wilsey B. J. (2017). Species composition but not diversity explains recovery from the 2011 drought in Texas grasslands. Ecosphere 8, e01704. doi: 10.1002/ecs2.1704

[B83] Yang Y. Li T. Pokharel P. Liu L. Qiao J. Wang Y. . (2022). Global effects on soil respiration and its temperature sensitivity depend on nitrogen addition rate. Soil Biol. Biochem. 174, 108814. doi: 10.1016/j.soilbio.2022.108814

[B84] You C. Wu F. Gan Y. Yang W. Hu Z. Xu Z. . (2017). Grass and forbs respond differently to nitrogen addition: a meta-analysis of global grassland ecosystems. Sci. Rep. 7, 1563. doi: 10.1038/s41598-017-01728-x 28484219 PMC5431500

[B85] Zhang M. Delgado-Baquerizo M. Li G. Isbell F. Wang Y. Hautier Y. . (2023). Experimental impacts of grazing on grassland biodiversity and function are explained by aridity. Nat. Commun. 14, 5040. doi: 10.1038/s41467-023-40809-6 37598205 PMC10439935

[B86] Zhao L.-P. Wang D. Liang F.-H. Liu Y. Wu G.-L. (2019). Grazing exclusion promotes grasses functional group dominance via increasing of bud banks in steppe community. J. Environ. Manage. 251, 109589. doi: 10.1016/j.jenvman.2019.109589 31546141

[B87] Zhao M. Running S. W. (2010). Drought-induced reduction in global terrestrial net primary production from 2000 through 2009. Science 329, 940–943. doi: 10.1126/science.1192666 20724633

[B88] Zheng Z. Bai W. Zhang W.-H. (2019). Clonality-dependent dynamic change of plant community in temperate grasslands under nitrogen enrichment. Oecologia 189, 255–266. doi: 10.1007/s00442-018-4317-x 30511091

[B89] Zhuang L. Wang J. Huang B. (2017). Drought inhibition of tillering in *Festuca arundinacea* associated with axillary bud development and strigolactone signaling. Environ. Exp. Bot. 142, 15–23. doi: 10.1016/j.envexpbot.2017.07.017

