# Supplementary material for: Nitrogen addition and drought impose divergent effects on belowground bud banks of grassland community: a meta-analysis
Source: Front Plant Sci. 2025 Jan 6;15:1464973. doi: 10.3389/fpls.2024.1464973 (PMC11742946; doi:10.3389/fpls.2024.1464973)
Supplement: Supplementary file 1 [file DataSheet1.docx]

Materials and Methods S1

List of published studies from which data were extracted. Drought, N addition, increased precipitation, wildfire, grazing, clipping, herbivory, buds, bud bank, bud density.

Benson, E. J., Hartnett, D. C., and Mann, K. H. (2004). Belowground bud banks and meristem limitation in tallgrass prairie plant populations. *Am. J. Bot.* 91(3), 416-421.

Bombo, A. B., Appezzato-daGlória, B, and Fidelis, A. (2022). Fire exclusion changes belowground bud bank and bud-bearing organ composition jeopardizing open savanna resilience. *Oecologia* 199, 153-164.

Bombo, A. B., Siebert, F., and Fidelis, A. (2022). Fire and herbivory shape belowground bud banks in a semi-arid African savanna. *Afr*. *J. Range For. Sci.* 1-11.

Busso,C., Boo, R. M., and Pelaez, D. V. (1993). Fire effects on bud viability and growth of Stipa tenuis in semiarid Argentina. *Ann. Bot.* 71, 377-381.

Busso, C. A., Gittins, C., and Becker, G. (2011). Tiller hierarchy and defoliation frequency determine bud viability in the grass *Poa ligularis*. *Ecol. Res.* 26, 985-997.

Busso, C. A., and Richards, J. H. (1989). Effects of drought and defoliation on bud viability in two caespitose grasses. *Ann. Bot.* 63, 477-485.

Busso, C. A. (1997). Effects of defoliating *Stipa tenuis* and *Piptochaetium napostaense* at different phenological stages: Axillary bud viability and growth. *J. Arid Environ.* 35, 233-250.

Carter, D. L., Vanderweide, B. L., and Blair, J. M. (2012). Drought-mediated stem and belowground bud dynamics in restored grasslands. *Appl. Veg. Sci.* 15, 470-478.

Chen, X. R., Zhu, L. L., and Deng, Z. M. (2020). Effects of clipping on bud bank and population regeneration of *triarrhena lutarioriparia* in Dongting Lake Wetland, China. *Wetlands* 40, 2635-2642.

Chen, X. S., Deng, Z. M., and Xie, Y. H. (2016). Consequences of repeated defoliation on belowground bud banks of *Carex brevicuspis (Cyperaceae)* in the Dongting Lake Wetlands, China. *Front. Plant Sci.* 7, 1119.

Cui, W., Zhao, L. P., and Zhao, F. R. (2017). Effects of fencing and grazing management on bud bank in a semiarid steppe on the Loess Plateau. *Pratacultural Science* 34(1), 9-15.

Dalgleish, H. J., and Hartnett, D. C. (2009). The effects of fire frequency and grazing on tallgrass prairie productivity and plant composition are mediated through bud bank demography. *Plant Ecol.* 201, 411-420.

Dalgleish, H. J., Kula, A. R., and Hartnett, D. C. (2008). Responses of two bunchgrasses to nitrogen addition in tallgrass prairie: The role of bud bank demography. *Am. J. Bot.* 95(6), 672-680.

Fidelis, A., Appezzato-da-Glóriab, B., and Pillarc, V. D. (2014). Does disturbance affect bud bank size and belowground structure diversity in Brazilian subtropical grasslands. *Flora* 209, 110-116.

Fidelis, A., Muller, S. C., and Pillar, V. D. (2010). Population biology and regeneration of forbs and shrubs after fire in Brazilian Campos grasslands. *Plant Ecol*. 211(1), 107-117.

Flemmer, A. C., Busso, C. A, and Fernandez, O. A. (2002). Bud viability in perennial grasses: Water stress and defoliation effects. *J. Range Manage.* 55, 150-163.

Hartnett, D. C. (1991). Effects of fire in tallgrass prairie on growth and reproduction of prairie coneflower (*Ratibidacolumnifera*: Asteraceae), *Am. J. Bot.* 78, 429-435.

Hendrickson, J. R, and Briske, D. D. (1997). Axillary bud banks of two semiarid perennial grasses: occurrence, longevity, and contribution to population persistence. *Oecologia* 110, 584-591.

Hiers, Q. A, Treadwell, M. L, and Dickinson, M. B. (2021). Grass bud responses to fire in a semiarid savanna system. *Ecol. Evol.* 11, 6620-6633.

Kammesheidt, L. (1999). Forest recovery by root suckers and above-ground sprouts after slash-and-burn agriculture, fire, and logging in Paraguay and Venezuela. *J. Trop. Ecol.* 15,143-157.

Lee, P. (2004). The impact of burn intensity from wildfires on seed and vegetative banks and emergent understory in aspen-dominated boreal forests. *Can. J. Bot.* 82, 1468-1480.

Li, Z. M., Wu, J. F., and Han Q. 2021. Nitrogen and litter addition decreased sexual reproduction and increased clonal propagation in grasslands. *Oecologia* 195, 131-144.

Luo, W., Muraina, T. O., Griffin-Nolan, R. J., Ma, W., Song, L., Fu, W.,and Collins, S. L. (2023). Responses of a semiarid grassland to recurrent drought are linked to community functional composition. *Ecology* 104, e3920.

Ott, J. P, Butler, J. L, and Rong, Y. P. (2017). Greater bud outgrowth of *Bromus inermis* than *Pascopyrum smithii* under multiple environmental conditions. *J. Plant Ecol.* 10, 518-527.

Pausas, J. G., Lamont, B. B., and Paula, S. (2018). Unearthing belowground bud banks in fire-prone ecosystems. *New Phytol.* 217, 1435-1448.

Pel´aez, D. V., Boo, R. M., and Elia, O. R. (1997). Effect of fire intensity on bud viability of three grass species native to central semi-arid Argentina. *J. Arid Environ.* 37, 309-317.

Qian, J. Q., Guo, Z. Y., and Muraina, T. O. (2022). Legacy effects of multi-year extreme drought on belowground bud banks in rhizomatous vs bunchgrass-dominated grasslands. *Oecologia* 198, 763-771.

Qian, J. Q., Wang, Z. W.,and Klimesova, J. (2021). Belowground bud bank and its relationship with aboveground vegetation under watering and nitrogen addition in a temperate semiarid steppe. *Ecol. Indic.* 125, 107520.

Qian, J. Q., Wang, Z. W,and Liu, Z. M. (2017). Belowground bud bank responses to grazing intensity in the Inner-Mongolia Steppe, China. *Land Degrad. Dev.* 28, 822-832.

Qian, J. Q., Zhang, Z., Dong, Y., Ma, Q., Yu, Q., Zhu, J.,and Luo, W. (2023). Responses of bud banks and shoot density to experimental drought along an aridity gradient in temperate grasslands. *Funct. Ecol.* 37, 1211-1220.

Russell, M. L.,and Vermeire, L. T. (2015). Fire and nitrogen alter axillary bud number and activity in purple threeawn. [*Rangeland Ecol. Manag.*](javascript:void(0)) 68, 65-70.

Russell, M. L., Vermeire, L. T.,and Ganguli, A.C. ( 2015). Season of fire manipulates bud bank dynamics in northern mixed-grass prairie. *Plant Ecol*. 216, 835-846.

Russell, M. L., Vermeire, L. T.,and Ganguli, A. C. (2019). Fire return interval and season of fire alter bud banks. [*Rangeland Ecol. Manag.*](javascript:void(0)) 72, 542-550.

VanderWeide, B. L., Hartnett, D. C.,and Carter, D. L. (2014). Belowground bud banks of tallgrass prairie are insensitive to multi-year, growing-season drought. *Ecosphere* 5(8), 103.

VanderWeide, B. L.,and Hartnett, D. C. (2015). Belowground bud bank response to grazing under severe, short‑term drought. *Oecologia* 178, 795-806.

Wang, J. F., Shi, Y. J.,and Ao, Y,N. ( 2019). Summer drought decreases *Leymus chinensis* productivity through constraining the bud, tiller and shoot production. *J. Agron. Crop Sci.* 205, 554-561.

Wang, J. Y., Xu, T. T.,and Feng, X. Y. (2022). Simulated grazing and nitrogen addition facilitate spatial expansion of *Leymus chinensis* clones into saline-alkali soil patches: Implications for Songnen grassland restoration in northeast China. *Land Degrad. Dev.* 33, 710-722.

Wang, Q., Guo, Z. G.,and Pang, X. P. (2020). Effects of small-herbivore disturbance on the clonal growth of two perennial graminoids in alpine meadows. *Alpine Bot.* 130, 115-127.

Wang, Q., Yu, C.,and Pang, X. P. (2018). The disturbance and disturbance intensity of small and semi-fossorial herbivores alter the belowground bud density of graminoids in alpine meadows. *Ecol. Eng.* 113, 35-42.

Xing, Y. (2019). Effects of nitrogen application on the yield, quality and bud bank of mixed planting of *Leymus chinensis* and *Lespedeza daurica*. Northeast Normal University.

Yuan, J. H., Wang, P.,and Yang, Y. F. (2019). Effects of simulated herbivory on the vegetative reproduction and compensatory growth of *hordeum brevisubulatum* at different ontogenic stages. *International Journal of Environmental Research and Public Health* 16, 1663.

Zhang, C. Y. (2014). Responses of bud bank and sexual reproduction of main plants to stimulated precipitation and nitrogen deposition in *Stipa krylovii* steppe. Northeast Normal University.

Zhang, J.F., Li, Q. H. ,and Wang, L.L. (2018). Effects of different nitrogen addition on bud bank traits and branching architecture of *nitraria tangutorum* seedlings. *Forest Res.* 31(3), 158-166.

Zhang, J. F. (2017). Responses of bud bank dynamics of *nitraria tangutorum* to nitrogen addition. Chinese Academy of Forestry Sciences.

Zhao, L. P.,Wang, D.,and Liang, F.H. (2019). Grazing exclusion promotes grasses functional group dominance via increasing of bud banks in steppe community. *J. Environ. Manage.* 251, 109589.

Zhao, P., Cui, W.,and Bai, X. (2017). The bud banks in the typical steppe communities with different disturbance regimes. *Russ. J. Ecol.* 48, 551-558.

**Table S1** The detail information for the 46 publications.

| Country | Ecosystem | Moderator | Plant group | Bud type | Basis for classification | Degree/Amount/Concentration | Effect/observation | Reference |
| --- | --- | --- | --- | --- | --- | --- | --- | --- |
| Southern Brazil | Grassland | Fire | Ungrouped grass  shrub | Ungroup bud | Number of fire | 1 | The study showed a decrease in bud bank size in the absence of fire. | Fidelis et al., 2014 |
| Kansas, USA | Tallgrass Prairie | Fire | Ungrouped  grass  shrub | Rhizome bud | Number of fire | 3 | The bud bank density in the unburned prairie was significantly lower than aboveground stem densities. | Benson et al., 2004 |
| South African | Tropical savannas | Fire | Grass, forb, shrub | Ungroup bud | Number of fire | 1, 3, 7, 16, 20 | Lower fire frequency was expected to reduce the bud bank density. | Bamboo et al., 2022 |
| South African | Tropical savannas | Fire | Ungrouped | Ungroup bud | Number of fire | 1, 3, 7, 16, 20 | The total bud bank increased at a lower frequency. | Bamboo et al., 2022 |
| Paraguay | Forest | Fire | Ungrouped | Ungroup bud | Number of fire | 1 | In the fire-degraded stand, the density of resprouts increased. | Kammesheidt, 1999 |
| China | Typical steppe grassland | Fire | Ungrouped | Rhizome bud  tiller bud ungroup bud | Number of fire | 1 | Fire significantly increased bud bank density. | Zhao et al., 2017 |
| Argentina | Forage grass | Fire | Grass | Ungroup bud active bud  dormant bud, dead bud | Number of fire | 1 | Controlled burning with high fuel loads can severely reduce bud viability in S.tenuis. Plants located in areas of lighter fuel accumulation. | Busso et al., 1993 |
| Texas, USA | Semi-arid savanna | Fire | Grass shrub | Ungroup bud active bud  dormant bud  dead bud | Fire frequency | Low, High | Fire energy directly affects bud activity and mortality through soil heating for these two species. | Hiers et al., 2020 |
| La Pampa, Argentina | Perennial grasses | Fire | Grass | Ungroup bud active bud dormant bud dead bud | Number of fire | Low, High | Tillers of plants exposed to fire had more dead buds and fewer metabolically active buds than the tillers of control plants. | Pelaez et al., 1997 |
| Montana, USA | Semi-arid mixed grass prairie | Fire | Grass | Active bud, dormant bud | Number of fire | 1 | Fall and summer fires reduced total buds per tiller by about 70%. | Russell and Vermeire, 2014 |
| Montana, USA | Semi-arid mixed grass prairie | Fire | Grass | Active bud, dormant bud, dead bud | Number of fire | 1 | Season of fire directly manipulated bud activity, dormancy, and mortality. | Russell et al., 2015 |
| Montana, USA | Semi-arid mixed grass prairie | Fire | Grass | Active bud | Number of fire | 1 | H. comata bud mortality increased immediately following the prescribed fires for summer and fall. | Russell et al., 2019 |
| Southern Brazil | Grassland | Fire | Forb  shrub | Ungroup bud | Fire frequency | High | The bud bank of E. ligulaefolium tended to be larger in excluded sites. V. flexuosa showed a larger bud bank in frequently burned sites. | Fidelis et al., 2010 |
| Kansas, USA. | Konza prairie | Fire | Forb | Ungroup bud | Fire frequency | Low | Plants from sites not burned for many years produced 50% more stems than plants from recently burned sites. | Hartnett, 1991 |
| Alberta, Canada | boreal forest | Fire | Grass | Ungroup bud | Fire frequency | Low, High | Vegetative bank assemblages significantly differed between unburned and burned patches but not between lightly and intensely burned patches. | Lee, 2004 |
| Kansas, USA | Perennial grasses | Grazing | Grass | Ungroup bud | Livestock number | 0.06/ha | Grazing decreased grass bud banks compared to ungrazed prairie. | Dalgleish and Hartnett, 2009 |
| Inner Mongolia, China | Perennial bunchgrass | Grazing | Grass | Ungroup bud rhizome bud tiller bud  root sprouting bulb bud | Livestock number | 6/ha, 12/ha, 18/ha, 24/ha, 30/ha, 36/ha | The belowground bud bank seems to be tolerant of grazing. | Qian et al., 2017 |
| Inner Mongolia, China | Perennial bunchgrass | Grazing | Grass | Rhizome bud tiller bud  ungroup bud | Livestock number | 6/ha, 12/ha, 18/ha, 24/ha, 30/ha, 36/ha | Species that rely on rhizomes and bulb buds for population regeneration are more sensitive to grazing. | Qian et al., 2017 |
| Loess Plateau, China | Temperate grassland | Grazing | Ungrouped  grass  forb | Ungroup bud | Livestock number | 2-3.5/ha | Long-term grazing exclusion significantly increased plant buds and bud bank size. | Zhao et al., 2019 |
| Loess Plateau, China | Typical steppe | Grazing | Ungrouped | Rhizome bud tiller bud root sprouting bud | Livestock number | 2-3.5/ha | Grazing significantly decreased tiller bud bank density. | Zhao et al., 2017 |
| Loess Plateau, China | Grassland | Grazing | Ungrouped  grass  forb | Rhizome bud tiller bud  root sprouting bud  ungroup bud | Livestock number | 4.6/ha | Compared with grazing, short-term closure significantly increased the total bud bank density and the density of grass bud bank but significantly decreased the density of non-grass bud bank. | Cui et al., 2017 |
| Kansas, USA. | Tallgrass prairie | Grazing | Grass  forb | Ungroup bud | Clipping ration | 42% | Grazing affects their (buds and rhizomes) growth and survival. | VanderWeide and Hartnett, 2015 |
| Northeastern, China | Grassland | Grazing | Ungrouped | Ungroup bud | Clipping ration | 40% | Simulated moderate grazing increased the bud density of ramets across the years by 52%. | Wang et al., 2021 |
| Dongting Lake, China | Wetland | Grazing | Grass | Ungroup bud, tiller bud | Clipping ration | Low, High | Compared with the control, bud bank density decreased significantly under HSC. | Chen et al., 2020 |
| Dongting Lake, China | Wetland | Grazing | Grass | Rhizome bud | Clipping ration | Low | The density and biomass of rhizome buds did not decrease significantly in response to repeated defoliation. | Chen et al., 2016 |
| Texas, USA | Semiarid perennial grasses | Grazing | Grass | Active bud, dormant bud | Livestock number | High | The grazing history of the communities from which the buds were collected did not substantially affect the number of axillary buds. | Hendrickson and Briske, 1997 |
| Kansas, USA. | Tallgrass prairie | Grazing | Grass  forb | Ungroup bud | Clipping ration | 42% | Grazing reduced grass buds per shoot from approximately 7.5 to 5 buds per shoot. | VanderWeide and Hartnett, 2015 |
| South Dakota, USA | Grassland | Grazing | Grass | Ungroup bud | Clipping ration | Mediate | Clipped tillers had significantly lower numbers of propagules than unclipped tillers. | Ott et al., 2017 |
| Northeastern, China | Grassland | Grazing | Grass | Ungroup bud | Clipping ration | 50% | Moderate clipping intensities significantly increased the total number of juvenile tillers and buds. | Yuan et al., 2019 |
| Argentina | Perennial bunchgrass | Grazing | Grass | Ungroup bud active bud  dormant bud, dead bud | Clipping ration | 50% in 1, 2, 3, 4 or 5 defoliations | The total number of axillary buds per stem base was similar in all defoliation frequencies. | Busso et al., 2011 |
| Argentina | Perennial bunchgrass | Grazing | Grass | Ungroup bud active bud dormant bud dead bud | Clipping ration | Mediate | Plants of both species that have defoliated late or after internode elongation had a greater number of respiratory inactive buds than undefoliated plants. | Busso et al., 1997 |
| Utah, USA | Bunch grass | Grazing | Grass | Ungroup bud | Clipping ration | 85% | Long periods of defoliation resulted in reduced tiller number, probably by causing inactivation of axillary buds. | Busso et al., 1989 |
| Inner Mongolia, China | Steppe | Grazing | Grass  forb | Ungroup bud | Clipping ration | Mediate | Mowing had no significant effect on bud number. | Zhang, 2014 |
| Qinghai-Tibetan Plateau, China | Alpine Meadows | Grazing | Grass | Rhizome bud tiller bud ungroup bud | Clipping ration | Low | This study showed that disturbance by plateau pikas increased tiller bud number and rhizome bud number per clonal fragment. | Wang et al., 2020 |
| China | Alpine Meadows | Grazing | Ungrouped grass | Rhizome bud tiller bud | Herbivore number | Low | Disturbance by the plateau pika increased the belowground bud density of graminoids but did not affect forbs bud density. | Wang et al., 2018 |
| Inner Mongolia, China | Temperate semiarid steppe | N addition | Ungrouped | Rhizome bud tiller bud roots prouting  bulb bud ungroup bud | Amount of N addition | 10g/m2 | In addition, it decreased total bud density but facilitated aboveground productivity, and buds of grasses and forbs responded in the opposite way. | Qian et al., 2021 |
| China | Degraded grasslands | N addition | Grass | Ungroup bud | Amount of N addition | 10g/m2 | N addition increased the bud density of Leymus chinensis ramets by 98 %. | Wang et al., 2021 |
| Inner Mongolia, China | Steppe | N addition | Ungrouped | Rhizome bud tiller bud root sprouting bulb bud ungroup bud | Amount of N addition | 10g/m2 | N addition increased the density of the root sprouting bud and decreased the density of the bulb bud. | Zhang, 2014 |
| Inner Mongolia, China | Desert | N addition | Shrub | Ungroup bud dormant bud | Amount of N addition | 12mmol/L, 24mmol/L,36mmol/L,48mmol/L,60 mmol/L | N addition significantly increased the number of total buds and significantly reduced the number of dormant buds. | Zhang et al., 2018 |
| Inner Mongolia, China | Desert | N addition | Shrub | Ungroup bud dormant bud | Amount of N addition | 12g/m2,24g/m2,49g/m2,73g/m2,98g/m2, 122g/m2 | Nitrogen addition significantly increased the bud number of Nitraria tangutorum seedlings and significantly decreased the number of dormant buds. | Zhang, 2017 |
| Inner Mongolia, China | Steppe | N addition | Grass  forb  shrub | Ungroup bud | Amount of N addition | 10g/m2 | N addition increased the tiller bud density of Stipa krylovii and the root sprouting bud of Potentilla tanacetifolia. | Zhang, 2014 |
| China | Grassland | N addition | Grass  forb | Rhizome bud tiller bud ungroup bud | Amount of N addition | 10g/m2 | N addition increased the total bud density of Leymus chinensis by 284.22% and Lespedeza daurica by 57.55%. | Xing, 2019 |
| Kansas, USA. | Tallgrass prairie | N addition | Grass | Ungroup bud | Amount of N addition | 10g/m2 | Nitrogen addition significantly impacted bud bank demography in two subdominant species. | Dalgleish et al., 2009 |
| Kansas, USA. | Tallgrass prairie | Drought | Grass | Ungroup bud | Amount of rainfall intercepted | 886mm | Bud bank density was insensitive to drought. | VanderWeide et al., 2014 |
| Kansas, USA. | Tallgrass prairie | Drought | Grass  forb | Ungroup bud | Amount of rainfall intercepted | 408mm | drought reduced the bud bank density of all taxonomic groups. | VanderWeide and Hartnett, 2015 |
| south-central Nebraska, USA | Restored grasslands | Drought | Grass  forb | Ungroup bud | Amount of rainfall intercepted | 629mm | Drought reduced below-ground bud bank density. However, bud bank density recovered, and bud production was higher on previously droughted subplots relative to controls in the year following drought. | Carter et al., 2012 |
| Inner Mongolia, China | Grasslands | Drought | Grass | Ungroup bud | Proportion of rainfall intercepted | 66% | drought reduced belowground bud density. However, drought had no significant influence on belowground buds of the dominant plant growth form in each community. | Qian et al., 2022 |
| Kansas, USA. | Tallgrass prairie | Drought | Grass  forb | Ungroup bud | Amount of rainfall intercepted | 408mm | grass bud density remained constant across all drought treatments. Meanwhile, sedge and forb bud density was reduced by drought. | VanderWeide and Hartnett, 2015 |
| Kansas, USA. | Tallgrass prairie | Drought | Grass | Ungroup bud | Amount of rainfall intercepted | 886mm | The number of buds per shoot did not differ among treatments. | VanderWeide et al., 2014 |
| Utah, USA | Bunch grass | Drought | Grass | Ungroup bud | Amount of rainfall intercepted | 368mm, 555mm, 675mm | 6-33% (mean=22%) of the bud pool was active in the drought treatment. | Busso et al., 1989 |
| Bahia Blanca, Argentina | Perennial grass | Drought | Grass  forb | Ungroup bud active bud dormant bud | Amount of rainfall intercepted | 200mm | Axillary bud activation is lower under water stress than under higher soil moisture conditions in Stipa clarazii, Stipa tenuis, and Stipa gynerioides. | Flemmer et al., 2002 |
| Northeastern, China | Perennial grass | Drought | Grass | Rhizome bud tiller bud | Proportion of rainfall intercepted | 50%, 70% | Drought decreased the bud bank density by 56%. In addition, drought  induced a bud allocation change that decreased by 41% the proportion of buds that developed into shoots and a 41% increase in the buds that developed into rhizomes. | Wang et al., 2019 |
| Northern, China | Temperate grassland | Drought | Ungrouped  Grass  forb | Ungrouped | Proportion of rainfall intercepted | 66% | Belowground bud density was lowest at the highest aridity site for the entire community. Belowground bud density was the lowest for grasses at the high aridity site but the highest for forbs at this site. | Qian et al., 2023 |
| Northern, China | Temperate grassland | Drought | Ungrouped | Ungrouped | Proportion of rainfall intercepted | 100% | Drought increased tiller abundance in the first treatment year and reduced bud banks by the fourth treatment year across grasslands. | Luo et al., 2023 |

Table S2 Basis for the classification of the degree of each moderator.

| Moderator | Basis of ranking | Low | Mediate | High |
| --- | --- | --- | --- | --- |
| Wildfire | Number of wildfires | ≤5 | 5-10 | ≥10 |
| Grazing | Livestock per hectare  Clipping proportion | ≤10/ha  ≤30% | 10-30/ha  30-50% | ≥30/ha  ≥50% |
| N addition | Amount  Concentration | ≤20g/m^2^  ≤ 20 mmol/L^-1^ | 20-50g/m^2^  20-40mmol/L^-1^ | ≥50g/m^2^  ≥40mmol/L^-1^ |
| Drought | Amount of rainfall intercepted  Proportion of rainfall intercepted | ≤ 200mm  ≤30% | 200-500 mm  30-60% | ≥ 500 mm  ≥60% |
